# Mechanically stable polymer networks incorporating polymeric ionic liquids for enhanced conductivity in solid-state electrolytes

**DOI:** 10.1080/15685551.2024.2449444

**Published:** 2025-01-07

**Authors:** Sezer Özenler, Nataliya Kiriy, Upenyu L. Muza, Martin Geisler, Anton Kiriy, Brigitte Voit

**Affiliations:** a Leibniz-Institut für Polymerforschung Dresden e.V, Dresden, Germany; b beeOLED GmbH, Dresden, Germany; c Organic Chemistry of Polymers, Technische Universität Dresden, Dresden, Germany

**Keywords:** Polymeric ionic liquids (PILs), solid-state electrolytes, cross-linked polymer networks, ionic conductivity, lithium batteries

## Abstract

Enhancing both ionic conductivity and mechanical robustness remains a major challenge in designing solid-state electrolytes for lithium batteries. This work presents a novel approach in designing mechanically robust and highly conductive solid-state electrolytes, which involves ionic liquid-based cross-linked polymer networks incorporating polymeric ionic liquids (PILs). First, linear PILs with different side groups were synthesized for optimizing the structure. Molecular weights of the PIL samples, ranging from 30 to 40 kDa, were determined using a complimentary combination of thermal field-flow fractionation (ThFFF) and matrix-assisted laser desorption/ionization time-of-flight mass spectrometry (MALDI-TOF MS) analysis. The aimed for networks were synthesized through the photo-initiated polymerization of a network-forming monomer and a cross-linker, in the presence of lithium bis(trifluoromethanesulfonyl)imide (LiTFSI) and a PIL bearing quaternized imidazolium groups. The resulting cross-linked membranes – semi-interpenetrating networks – exhibit substantial mechanical strength, with a Young’s modulus of 40–50 MPa, surpassing the threshold for solid-state battery separators, while maintaining high ionic conductivity in the range of 4 × 10^−4^ S·cm^−1^ at 60°C. Notably, the introduction of oligo(ethylene glycol) moieties into the PIL structure significantly enhances ionic conductivity and allows for incorporation of a larger amount of the lithium salt compared to the alkyl-substituted analogs. Moreover, although cross-linking often impairs ionic transport as a result of restricted segmental mobility of the polymer chains, incorporation into the network of highly conductive linear PILs circumvents this issue. This unique combination of properties positions the developed membranes as promising candidates for application in solid-state lithium batteries, effectively addressing the traditional trade-off in electrolyte design.

## Introduction

Li-ion batteries are a promising alternative energy source for replacing gasoline in automotive applications, particularly for pure electric and hybrid vehicles as well as in various electronic devices [1]. However, several challenges hinder the widespread adoption of rechargeable lithium-metal batteries (LMBs) [2]. One of the most significant issues is the inhomogeneous electrodeposition of lithium, leading to the formation of high-surface-area lithium, primarily in the form of dendrites [3,4]. These dendrites pose serious safety concerns, especially when highly flammable organic liquid electrolytes are used, as they can penetrate the separator and cause internal short circuits [5]. Consequently, there is an urgent need to develop alternative electrolytes with high ionic conductivity, as well as improved mechanical, chemical, electrochemical, and thermal stability, to facilitate the large-scale adoption of LMBs.

Unlike conventional lithium-ion batteries, which utilize volatile and flammable liquid organic electrolytes, all-solid-state batteries employ solid electrolytes. This provides a safer solution for next-generation battery systems by suppressing dendrite growth and reducing the risk of combustion [6–8]. However, developing efficient all-solid-state lithium-ion batteries is a complex task. One of the key challenges is creating solid polymer electrolytes (SPEs) that combine adequate mechanical properties with high ionic conductivity [9,10]. In liquid Li-ion batteries, ions can move freely through the electrolyte solution, which promotes high ionic conductivity. In contrast, in SPEs, lithium ions are coordinated with segments of the polymer chains, and their diffusion is strongly dependent on the dynamics of the polymer backbone. While melting the polymer enhances the ionic conductivity, the conductivity drops significantly when the material is in a solid state. Typically, the ionic conductivity in liquid Li-ion batteries is at least two orders of magnitude higher than that of all-solid-state polymer electrolytes [11–13].

Polyethylene oxide (PEO)-based electrolytes, complexed with Li salts, have been extensively studied for over four decades as one of the most promising components for SPEs due to their favorable characteristics, such as high salt-complexing capacity, good corrosion resistance, mechanical flexibility, and chemical stability [14–18]. Some PEO/Li bis(trifluoromethanesulfonyl) imide (LiTFSI) complexes can achieve ionic conductivities of up to 10^− 3^ S·cm^− 1^ at 80°C. However, at ambient temperature, the conductivity drops below 10^− 7^ S·cm^− 1^ due to PEO’s tendency to crystallize [19]. This unfavorable temperature dependence is largely attributed to the fact that lithium-ion transport mainly occurs along the PEO chains, which is significantly aided by the segmental mobility of the polymer chains. When PEO crystallizes, this mobility is hindered, restricting ion transport.

**Figure 1.**
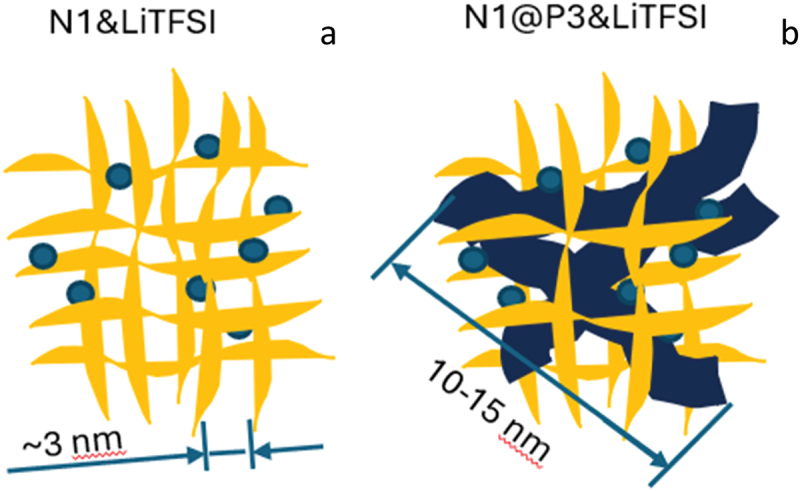
Schematic representation of the mechanically reinforced solid polymer electrolytes studied in this work: without (a) and with (b) entrapped poly(ionic liquid) chains. Characteristic lengths corresponding to linear polymer chain fragments primarily responsible for the ionic transport are indicated for each structure.

To mitigate the effects of crystallization, significant efforts have been made to achieve an amorphous state of PEO by employing complex polymer architectures, such as comb-like copolymers, random copolymers, block copolymers, and cross-linked networks, or by using polymer blends and plasticizers [20,21]. Ionic liquids (ILs), such as imidazole or pyrrolidine derivatives, quaternized with short-alkyl halides, have emerged as promising plasticizers due to their ultra-low vapor pressure, broad electrochemical stability window, high thermal and chemical stability, and non-flammability. For example, PEO/Li salt/IL ternary solid polymer electrolytes can achieve ionic conductivities of up to 10^−3^ S·cm^− 1^ at 80°C with pyrrolidinium-based ILs [22]. However, plasticizers, including IL-based ones, often adversely affect the mechanical properties of SPEs [20–22]. Polymeric ionic liquids (PILs) are another attractive class of electrolytes, offering high ionic conductivities. Similar to PEO-based systems, it remains a challenge to balance high ionic conductivity with desirable mechanical properties in a single PIL material [23–29]. Various strategies, such as employing comb-like copolymers or block copolymers, have been explored to address this challenge. For instance, Watanabe et al. [30] developed membranes composed of electrospun PEO-grafted-polyimide (PI-g-PEO) nanofibers complexed with LiTFSI, which achieved a conductivity of 10^−4^ S·cm^− 1^ at room temperature and a high elastic modulus of 93 MPa. Our group has recently reported the preparation of PIL membranes mechanically reinforced with polypropylene carbonates using a polymer blend approach [31]. Rolland et al. presented a block copolymer electrolyte incorporating a polystyrene block that forms self-standing, glassy nano-domains, achieving conductivities up to 10^−5^ S·cm^− 1^ at room temperature [32]. While these approaches demonstrate the potential for high mechanical strength and conductivity, the complexity of synthesizing block copolymers limits their industrial scalability. A more straightforward method for enhancing the mechanical properties of polymer electrolytes is through cross-linking, which forms covalent bonds between polymer chains to create robust networks. For example, our group previously reported the synthesis of imidazolium-containing PIL networks via cross-linking photopolymerization [33]. Cross-linking significantly improved the mechanical stability of the PIL samples, allowing the formation of free-standing membranes. However, this improvement in mechanical properties came at the cost of reduced ionic conductivity, likely due to disruptions in the extended conductive pathways by limiting the segmental mobility responsible for efficient ion transportation (Figure 1a) [34].

In an effort to address both conductivity and mechanical strength in a cost-effective manner, we designed a three-dimensional polymer framework by photo-initiated polymerization a cross-linker and a network-forming monomer in the presence of a linear PIL. During cross-linking, the linear chains were trapped within the network (Figure 1b). We assumed that the high conductivity inherent to the linear PIL will be also preserved in the network composite thus improving its ion-transportation ability. To further enhance compatibility between the polymer and monomer phases, as well as improve ion conductivity, a monomer bearing ionic-liquid pendant group was utilized as the network-forming component. Additionally, Li-ion-coordinating oligo(ethylene glycol) moieties were incorporated into the acrylate-based PIL structure instead of alkyl groups, an approach that has proven effective in previous studies [22,35–37]. The present work focuses on the synthesis, characterization of new semi-interpenetrating networks, and the study of their electrochemical and mechanical properties.

## Materials and methods

### Materials

Acryloyl chloride (96%, Alfa Aesar, Ward Hill, MA, U.S.A.); 6-bromo-1-hexanol (>95%, TCI), 10-bromo-1-decanol (>95%, TCI), 1-ethylimidazole (>98%, TCI), magnesium sulfate (MgSO4, >98%, Sigma Aldrich), silver nitrate (AgNO3, 0.1 M, Sigma Aldrich), triethylamine (TEA, 99%, Alfa Aesar), α,α’-azobis(isobutyronitrile) (AIBN), 1-(2-bromoethoxy)-2-(2-methoxyethoxy)ethane (>96%, TCI), imidazole (99%, Sigma Aldrich), and 2-[2-(2-chloroethoxy)ethoxy]ethanol (>96%, TCI) were used as received. Lithium bis(trifluoromethane sulfonyl)imide (LiTFSI, 99%, IoLiTec Ionic Liquids Technologies GmbH, Heilbronn, Germany) was dried under vacuum at 110°C for 24 h prior the use.

## Methods

### Preparation of the samples for electrochemical measurements

#### Pure PILs and bicomponent PIL/LiTFSI membranes

Pure PILs as well as bicomponent PIL/LiTFSI membranes were drop-casted inside Teflon rings and placed on the stainless-steel electrode of a Swagelok cell. The bi-component PIL/LiTFSI mixtures 10, 20, and 30 w% of LiTFSI were prepared by mixing of respective mono-component solutions for 6 h at 50°C. After the drop-casting, the samples were extensively dried at 100°C in vacuum for 12 h.

### Electrochemical measurements

All electrochemical measurements were performed by Gamry potentiostat, Interface 1010. For different electrochemical methods, Swagelok cells (Swagelok Co., Solon, OH, U.S.A.) were utilized having the following setups: symmetrical cell setup (Li0/PIL/Li0) for complex electrochemical impedance spectroscopy (EIS) and for potentiostatic polarization measurements (PPM); asymmetrical cell setup (steel/PIL/Li0), for plating-stripping experiments and for cyclic voltammetry (CV).

The potentiostatic impedance measurements were carried out with the following parameters: 1 MHz to 100 mHz at open-circuit voltage with 50 mV, AC current.

For pure PILs, which are liquids, the Teflon rings with height 0.1 cm and inner diameter 0.8 cm were used in EIS measurements to prevent short circuits, and the thicknesses of Teflon rings were used as the thickness of membrane.

The ionic conductivity was calculated by Equation (1),

(1) σ=d/RA

where d is the sample thickness, R is the bulk resistance, and A is the cross-sectional area of the sample.

The bulk resistance of PILs and membranes were read from the high-frequency intercept of the Nyquist plot with the Z’ real axis. The thicknesses of the PIL/LiTFSI membranes were determined by using Equation (2),

(2) A=W/d/S

where A is the thickness; W is the weight of the membrane in g; d is thedensity g/cm^3^, and S is the area in cm^2^.

The experimentally determined value of the density for PIL is 1.1 g/cm^3^. The calculated areas are 0.785 cm^2^ and 0.502 cm^2^ for the samples without and with Teflon rings, respectively.

The transference number (*t*_*+*_) for Li ions was determined by Bruce–Vincent potentiostatic polarization method. Potentiostatic polarization experiments were done with an applied voltage of 10 mV and polarization time of 40,000 s. The transference number for the Li+ ions was calculated according to Equation (3):

(3) $$t+=\mathrm{Iss}\left(\Delta V-\mathrm{IoRo}\right)/\mathrm{Io}\left(\Delta V-\mathrm{IssRss}\right)$$

where *I*_*ss*_ is the steady state current, *I*_*0*_ is the initial current, ΔV is the applied potential, *R*_*ss*_ *and R*_*0*_ are the electrode resistances before and after polarization, respectively. *R*_*ss*_ and *R*_*0*_ were determined by fitting model parameters with a suitable equivalent circuit using Gamry Echem Analyst software, simplex method.

### Instrumentation

1H  nuclear magnetic resonance (NMR) spectra were recorded on Avance III 500 Spectro-meter (Brucker Corp. Billerica, MA, U.S.A.) at ambient temperature. The dimethyl sulfoxide (DMSO-d_6_) was used as a solvent.

Thermogravimetric analyses (TGA) were carried out on a Q5000 (TA Instruments, Newcastle, DE, U.S.A.) under nitrogen at a heating rate of 10 K⋅min^−1^ in the temperature range from 30°C to 800°C.

Differential scanning calorimetry (DSC) measurements were performed on a DSC 2500 (TA Instruments, Newcastle, DE, U.S.A.) under nitrogen with heating and cooling rates of 10 K⋅min^−1^. The PIL samples were measured by heating-cooling-heating cycles in the temperature range of −120°C to 200°C. The bi- and tri-component membranes were measured in three following regimes: 1^st^ heating to 100°C – cooling − 2^nd^ heating to 200°C – cooling − 3^rd^ heating to 100°C.

ThFFF experiments on the linear PILs P1, P2, and P3 were carried out with a TF2000 system with the following channel dimensions: 45.6 cm tip-to-tip length, width 2 cm, 250 μm thickness and spacer material made from Mylar A and Teonex by DuPont Teijin Films Ltd. The auxiliary instrumentations were: an isocratic pump, degasser, auto-sampler, actively heated and cooled ThFFF channel, PN3621 21-multi angle light scattering (MALS) detector with a laser of the wavelength 532 nm, and PN3150 differential refractive index (dRI) detector (all by Postnova Analytics GmbH, Germany). The channel pressure was maintained between ≈ 10.0 to 12 Bar by installing a back-pressure tubing (inner diameter 0.001 mm) between the ThFFF channel and the MALS detector to avoid vaporization of the carrier solvent, and to stabilize the dRI detector by dampening pressure pulsations. The cold wall block of the channel was cooled by a liquid cooling circuit with a refrigeration unit Unichiller 025-MPC (2.5 kW) by Peter Huber Kältemaschinenbau GmbH (now AG), Germany. The samples were injected at 50 μL volumes for concentrations of ≈10 mg mL^−1^. All data recording and analysis were performed using the TF2000 version of the NovaFFF software (Postnova Analytics GmbH, Germany). ThFFF fractions were collected at peak maxima for subsequent analysis with MALDI-TOF MS, applied to determine the molar masses of the PIL*N,N*-Dimethylacetamide (DMAc) was used as carrier solvent and for sample dissolution.

MALDI-TOF mass spectra were recorded with a Bruker Autoflex Speed MALDI-TOF/TOF spectrometer, equipped with a Smartbeam-II Nd:YAG laser (355 nm, 1 kHz). 1,1-dicyano-4-(4’-tert-butylphenyl)-3-methyl-buta-1,3-diene (DCTB) was used as matrix. The collected liquid fractions from the ThFFF separation of unknown concentration were mixed in a ratio of 3 µL fraction solution to 7 µL of matrix solution (10 g/L, P1 and P2) or 4 µL to 6 µL (P3), respectively. Mass spectra were measured in linear positive TOF mode using a pulsed ion extraction delay of 1500 ns. Masses below m/z 9000 were suppressed by deflection. The TOF mode method was calibrated using BSA (2 M^+^ and M^+^ up to M^6+^) as calibrant.

Atomic force microscopy (AFM) measurements were performed on Dimension Icon, AFM (Bruker, U.S.A.). The topography study and E-Modulus determination were done in Tapping Mode TESPA and Peak Force QNM mode. All measurements were conducted at ambient condition.

### Results and discussion

**Figure 2.**
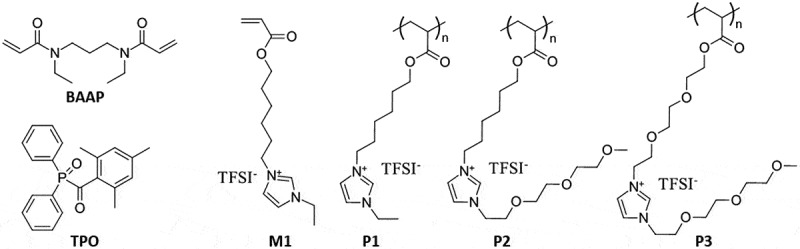
Chemical structures of the building blocks used in this study: a) BAAP – the cross-linker, b) TPO-initiator, c) M1 – the network-forming monomer, and three linear PILs as candidates for embedding into the polymer network: d) P1, e) P2, and f) P3.

Figure 2 illustrates the chemical structures of the acrylate-based building blocks used in this work: a) BAAP – a cross-linker, N,N’-diethyl-1,3-bis(acrylamide)-propane; b) TPO-initiator, diphenyl (2,4,6-trimethylbenzoyl)-phosphine oxide, c) M1 – a network-forming monomer containing quaternized imidazolium groups attached to the acrylate backbone via a hydrophobic hexyl group. Three linear polymers were selected as candidates for embedding into the polymer network: d) P1 – a PIL obtained by radical polymerization of M1; e) P2 – a linear PIL with an imidazolium group connected via a hexyl spacer (as in P1), but containing a tri(ethylene glycol) substituent instead of ethyl substituent; and f) P3 – a linear PIL in which both the connecting spacer and the substituent consist of tri(ethylene glycol) groups. Therefore, the content of ethylene glycol groups increases progressively from P1 to P2, and to P3.

**Figure 3.**
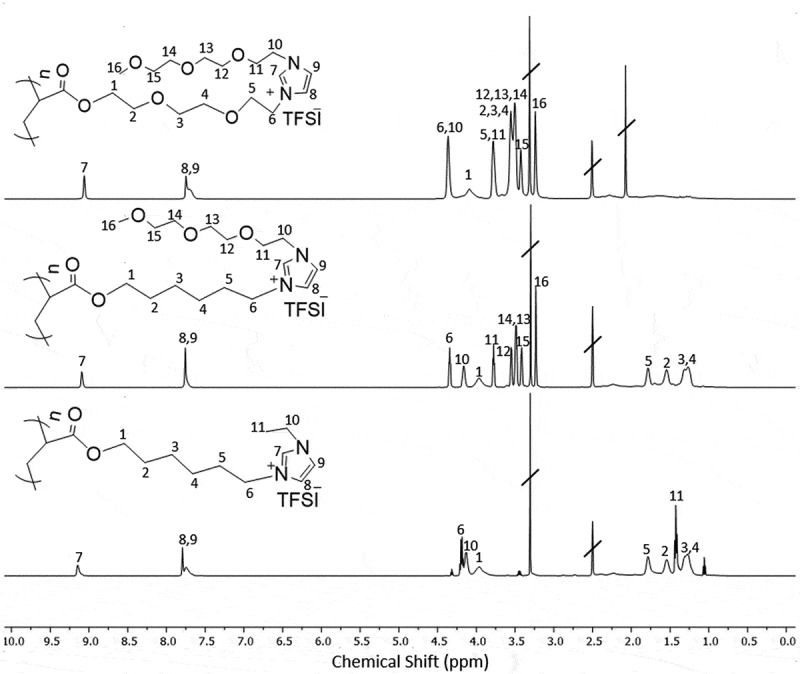
^1^ H NMR (DMSO-d₆) spectra of PILs P1, P2, and P3 with peak assignments. The spectra illustrate the characteristic signals of the polymers and their associated bis(trifluoromethanesulfonyl)imide (TFSI) counterions.

The incorporation of oligo(ethylene glycol) groups into the PILs was intended to enhance their interaction with Li cations. Recent studies have demonstrated that replacing hydrophobic alkyl substituents in IL plasticizers with cation-coordinating oligo(ethylene oxide) groups positively impacts Li+ ion transport by eliminating passive ‘diluents’ that hinder Li^+^ migration [33–36]. To determine the most suitable PIL for incorporation into the polymer network, P1, P2, and P3 were synthesized, and their physicochemical properties were evaluated. The synthesis of M1 has been previously described [28,29,31]. P1 was prepared through the free radical polymerization of M1. However, the synthesis of the tri(ethylene glycol)-based monomers encountered challenges, so PILs P2 and P3 were produced through polymer-analogous reactions (see Figure S2–S10, Supporting Information). For this, precursor polyacrylates containing halogen leaving groups at the ends of their side chains (Figure 3) were synthesized via free radical polymerization, and tri(ethylene glycol)-substituted imidazole groups were subsequently attached. The chemical identity of the polymers was confirmed unambiguously using ^1^H NMR spectroscopy. Notably, the spectra of P1, P2, and P3 feature signals characteristic of the attached groups, with intensities (from integrated spectra, not shown) indicating a near-complete degree of attachment of the tri (ethylene glycol)-substituted imidazole group (Figure 3).

The determination of molar mass for these polymers posed challenges due to interactions with the typical column materials used in size exclusion chromatography (SEC). To overcome this issue, we employed thermal field-flow fractionation (ThFFF), which utilizes an open-channel separation method and avoids such interactions. The hygroscopic nature of PILs makes it challenging to accurately evaluate the specific refractive index increment (dn/dc), an intrinsic parameter which is required to reliably determine molar mass using the conventional combination of the dRI and MALS. To resolve this, ThFFF was coupled offline with MALDI-TOF MS [29,31]. The molar masses obtained via MALDI-TOF MS after ThFFF are summarized in Table 1, showing a degree of polymerization in the 50–70 range (see Figure S11 and Table S1, Supporting Information).

**Table 1.** Average molar masses of PILs determined by ThFFF-MALDI-TOF MS and *T*_*g*_ of pure PILs, and ionic conductivities (σ) of pure PIL and mixtures with LiTFSI measured at RT and 60°C.

PIL	*M*_*n*_ [g·mol^−1^]	*T*_*g*_ [°C]	σ at RT/60°C [S·cm^−1^] PIL/LiTFSI: 0w/w%	σ at RT/60°C [S·cm^−1^] PIL/LiTFSI: 10 w/w%	σ at RT/60°C [S·cm^−1^] PIL/LiTFSI: 20 w/w%
P1	30700	−38	1.7 × 10^−5^/1.4 × 10^−4^	8.0×10^−6^/5.1 × 10^−5^	1.3×10^−6^/2.1 × 10^−5^
P2	36700	−49	1.8 × 10^−5^/2.0 × 10^−4^	1.1×10^−5^/3.9 × 10^−5^	1.0×10^−6^/2.0 × 10^−5^
P3	41800	−44	2.3 × 10^−5^/2.1 × 10^−4^	3.0×10^−6^/1.6 × 10^−4^	1.8×10^−6^/4.0 × 10^−5^

Thermal properties of the linear polymers were analyzed using differential scanning calorimetry (DSC) and thermogravimetric analyses (TGA). Glass transition temperatures (T_g_) were measured from the second heating cycle to eliminate the effects of residual solvents. As indicated in Table 1, the T_g_ values are notably low (−38°C and below), which is advantageous for ionic transport (see Figure S12, Supporting Information).

Thermal stability parameters, including the initial decomposition temperature (IDT), the temperature at which 10% weight loss occurs (T_d_10%), the temperature at which 50% weight loss occurs (T_d_50%), and the maximum decomposition rate temperature (MDT), were determined via TGA analysis (see Table S2, Supporting Information). The polymers P1, P2, and P3 show no signs of decomposition until 300°C, with IDT of ~300°C and T_d_10% of 350–380°C. Extensive decomposition begins above ~330°C, with T_d_50% occurring around 390°C and MTD of ~420°C. Thus, the PILs in this study demonstrate excellent thermal stability, far exceeding the minimum requirement of 150°C for lithium battery components [16,19].

**Figure 4.**
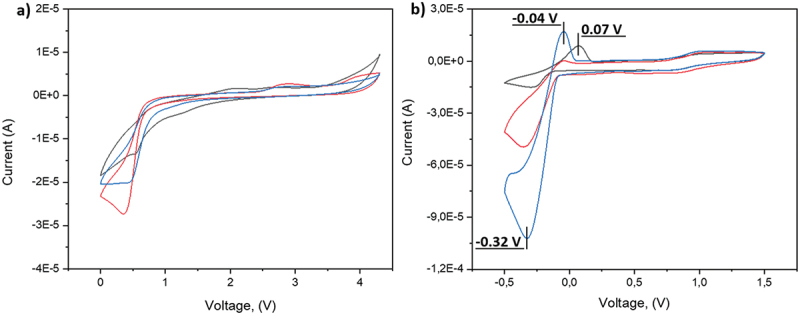
Cyclic voltammetry curves of (a) pure PILs: P1 (black), P2 (red), and P3 (blue); and (b) blends of P1, P2, and P3 with LiTFSI (10% w/w) at a scan rate of 1 mV/s and 60°C.

Figure 4a shows the cyclic voltammetry (CV) results of pure PILs – P1, P2, and P3 – indicating the absence of any significant reduction or oxidation reactions within the voltage range of +0.5 to +4 V vs. Li^+^/Li. Figure 4b presents the CV results at a scan rate of 1 mV/s and 60°C for P1, P2, and P3 after the addition of the conductive salt LiTFSI. The redox process in the 0 V range reflects lithium reduction at −0.3 V and oxidation at approximately 0 V vs. Li+/Li. Notably, for the mixtures of PILs and LiTFSI (Figure 4b) the transitioning from P1 to P2 and P3 leads to a threefold and sevenfold increase, respectively, in the reduction current.

**Figure 5.**
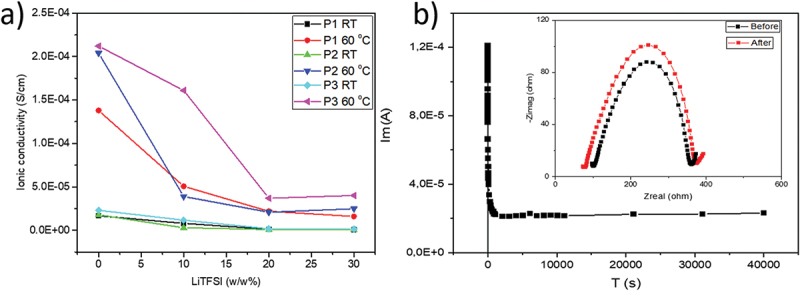
(a) Influence of added LiTFSI on the ionic conductivity of P1, P2, and P3 at room temperature (RT) and 60°C. (b) Time-dependent response of the dc polarization potential acquired on a Li/P3/Li cell at 60°C. The impedance spectra of P3 are shown before (black) and after (red) polarization in the inset, measured with a 0.1 mm thickness Teflon ring at 50 mV and with 20 mol% LiTFSI.

Figure 5a illustrates the relationship between the addition of LiTFSI and the ionic conductivity of PILs at room temperature (RT) and 60°C. In the absence of LiTFSI at 20°C, the ionic conductivity of all PILs was approximately 2.0 × 10^−5^ S·cm^−1^. At 60°C, without LiTFSI, the ionic conductivities increased to 1.4 × 10^−4^, 2.0 × 10^−4^, and 2.1 × 10^−4^ S·cm^−1^, for P1, P2, and P3, respectively (Table 1). Overall, an increase in LiTFSI content correlated with a decrease in ionic conductivity, which can be attributed to the increased viscosity of the PIL/LiTFSI blends. However, the effect varies among the different PILs: for P1 and P2, adding 10w/w% of LiTFSI (corresponding to approximately 20 mol% LiTFSI, see the values given in bold in Table 1 and Figure S19) resulted in a one-order-of-magnitude reduction in ionic conductivity, dropping to 5.1 × 10^−5^ S·cm^−1^ and 3.9 × 10^−5^ S·cm^−1^, respectively. In contrast, the ionic conductivity of P3 experienced only a slight decrease, from 2.1 × 10^−4^ S·cm^−1^ to 1.7 × 10^−4^ S·cm^−1^ (Figure S19). This phenomenon may be due to P3’s enhanced capacity for the Li^+^ cation, which arises from substituting alkane spacers with oligo(ethylene glycol) spacers.

Increasing the LiTFSI concentration from 0 to 30 w/w% led to a gradual decrease in ionic conductivity at 60°C, reaching a plateau in the 10–30 w/w% concentration range, for P1 and P2 and in the 20–30 w/w% for P3 (see Figure 5a). P1, which lacks oligo(ethylene glycol) moieties, reached a saturation point at a very low conductivity of less than 10^−6^ S·cm^−1^ (see Figures S13, Supporting Information). Notably, PILs containing ethylene glycol moieties (P2 and P3) achieved saturation at more significant levels of 3 × 10^−5^ S·cm^−1^ and 5 × 10^−5^ S·cm^−1^ for P2 and P3, respectively (see Figures S14–S17, Supporting Information). Consequently, P3 exhibited the best electrochemical properties among the studied polymers, making it the preferred candidate for further experiments aimed at enhancing mechanical properties while maintaining good ionic conductivity.

The conductivities presented above were evaluated using impedance spectroscopy, a technique that measures the overall ionic conductivities contributed by all ions in the system. To differentiate the contributions of various ions to the total ionic flux, the Bruce and Vincent potentiostatic polarization method was employed. This method involves applying a small constant direct current (dc) polarization potential to an electrolyte situated between non-blocking lithium electrodes for an extended period (greater than 10 h). During this polarization, the initial current (*Ii*) gradually decreases until it reaches a steady-state current (*Iss*). If no redox reactions involving the anions occur at the electrodes, the anion current will diminish in the steady-state regime. Because the polarization experiment is conducted near the electrochemical potential of lithium, the concentration of lithium cations remains constant, meaning that the steady-state current is representative of the lithium cations. The ratio of the steady-state current to the initial current (*Iss*/*Ii*) provides a rough estimate of the contribution of lithium cations to the overall ionic flux, which was found to be 0.19 for our solid polymer electrolyte containing P3 and 20 mol% LiTFSI (see Figure 5b, Supporting Information, Table S3). Notably, the original Bruce and Vincent formula for calculating a lithium transference number (t+), an important performance characteristic of electrolytes in lithium batteries, includes this *Iss/Ii* term along with a correction factor that accounts for the changes in resistance before and after polarization, as measured by impedance spectroscopy. However, the Bruce/Vincent method is limited to binary, ideal electrolytes, making it unsuitable for more complex solid-state electrolytes.

The application of the Bruce/Vincent method yields a t+ of 0.03, which is likely an underestimate. Previously, Atik et al. reported t+ values ranging from 0.05 to 0.1 for electrolytes containing ionic liquids with attached oligoethylene oxide chains, which enabled the development of efficient lithium batteries. The t+ obtained in our work aligns reasonably well with previously reported values; however, further improvements are needed.

**Figure 6.**
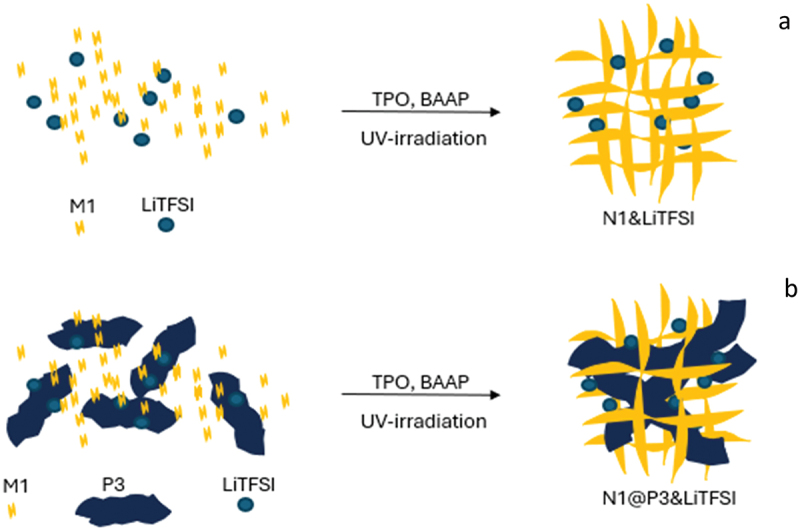
Schematic representation of the preparation process of the N1&LiTFSI and N1@P3&LiTFSI membranes.

We further explored the fabrication and properties of cross-linked SPEs. Figure 6 illustrates the preparation process of the cross-linked membranes, which involves photo-initiated polymerization of the network-forming monomer M1 with the cross-linker BAAP and LiTFSI, both in the absence and presence of the polymer ionic liquid P3. These formulations are denoted as N1×&LiTFSIy and N1×@P3z&LiTFSIy, respectively, with x, y, z indexes indicating the mole ratios of the components.

To prepare formulations #3 to #6 (as shown in Table 2), pure P3 (#4) or its mixtures with LiTFSI (#3, 5, and 6) were added to a blend of the monomer M1, the initiator TPO, and the cross-linking agent BAAP in the molar ratios specified in Table 2. UV irradiation was then applied to the Teflon mold. In formulation #6, an additional portion of LiTFSI was incorporated into the M1, TPO, and BAAP mixture, and a reduced amount of the cross-linker was used, specifically half the typical quantity. During the preparation process, as the UV light irradiated the mixtures, the monomer and cross-linker underwent polymerization to form a network that entrapped P3 and LiTFSI, as illustrated in Figure 6b.

**Table 2.** Molar content and ratios of the components in various N1@P3 formulations, and the corresponding mechanical integrity as well as the ionic conductivity of the resulting membranes.

#	Code	M1 [mol %]	TPO [mol %]	BAAP [mol %]	LiTFSI [mol %]	P3/ LiTFSI [mol %]	P3 [mol %]	Integrity	σ 60°C [S· cm^−1^]
0	P1	1	0.01	0	0	0:0	0	no	1.5x 10^−4^
1	N1	1	0.01	0.05	0	0:0	0	yes	1.2x 10^−4^
2	N1&LiTFSI_0.1_	1	0.01	0.05	0.1	0:0	0	yes	1.5x 10^−4^
3	N1@P3_0.13_&LiTFSI_0.08_	1	0.01	0.05	0	0.13: 0.08	0	yes	3.6x 10^−4^
4	N1@P3_0.15_	1	0.01	0.05	0	0:0	0.15	no	
5	N1@P3_0.26_&LiTFSI_0.16_	1	0.01	0.05	0	0.26: 0.16	0	yes	3.8x 10^−4^
6	N1_0.025_@P3_0.26_&LiTFSI_0.26_	1	0.01	0.05	0.1	0.26: 0.16	0	yes	4.0x 10^−4^

Formulations #0 to #3, which lack certain key components, were prepared for comparison to assess the impact of these components. Formulation #0, which does not include the cross-linker, polymer, or LiTFSI, corresponds to in-situ formed linear P1. Formulation #1 consists of a network formed from the monomer M1 and the cross-linker, effectively serving as an ‘empty container’ for formulations #3 to #6. Formulation #2 is similar to #1 but contains 10 mol% LiTFSI. These two formulations have been reported previously [33].

As expected, formulation #0, representing the non-crosslinked P1, is a viscous liquid that could not be peeled off from the Teflon mold, thus failing to form a free-standing membrane. Similarly, mixtures of in-situ forming P1 with LiTFSI, regardless of the ratios used, do not yield free-standing membranes, although the addition of LiTFSI increases the viscosity of the PILs, as previously shown [31].

The inclusion of the cross-linker in the formulations enhances the mechanical properties of the resulting materials, allowing for the successful production of good-quality membranes through the photoinduced polymerization of M1 and BAAP, with or without LiTFSI, resulting in N1 (#1) and N1&LiTFSI_0.1_ (#2), respectively. Notably, the incorporation of P3 into N1 (#4) significantly affected the mechanical properties, rendering it impossible to remove the membrane from the mold. In contrast, formulations #5 and #6, which contain both P3 and LiTFSI entrapped in the N1 network, exhibited mechanical stability. Remarkably, the reinforcing effect of LiTFSI was strong enough to maintain the integrity of the membrane produced with half the typical content of the cross-linker in formulation #6. In this case, a higher amount of LiTFSI was used. These results suggest that while polymer P3 acts as a plasticizer due to its liquid state, both BAAP and LiTFSI contribute to enhancing the stiffness of the materials.

**Figure 7.**
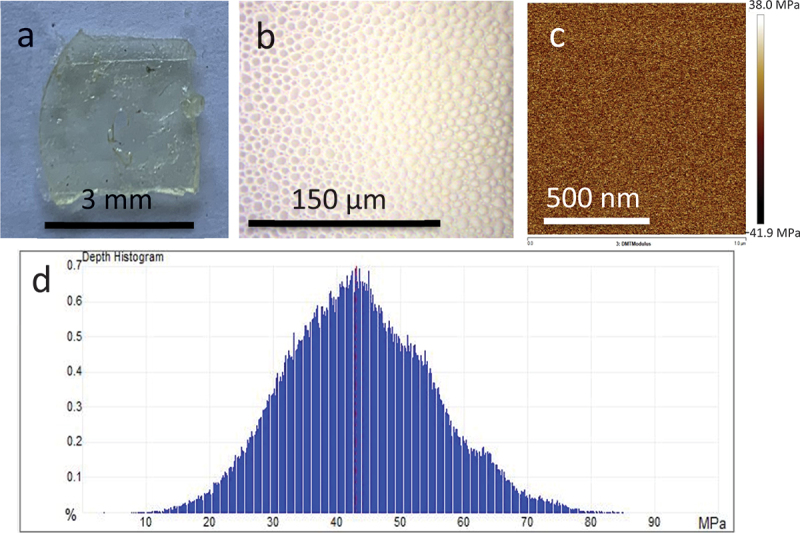
Photograph (a) and micrograph (b) of the free-standing crosslinked N1@P3_0.26_&LiTFSI_0.16_ membrane; young’s modulus image obtained by AFM QNM method (c) compiled into a histogram (d).

Figure 7a and b presents photographs and micrographs, respectively, of the free-standing crosslinked N1@P3_0.26_&iTFSI_0.16_ membrane (#5), which was prepared on a Teflon mold and subsequently peeled off the substrate using tweezers. The surface of the M1@P3 film appears homogeneous, but it features circular bumps a few micrometers in diameter. These bumps may be attributed to gaseous by-products generated from the peroxide initiator TPO.

The mechanical properties of the N1@P3_0.26_&LiTFSI_0.16_ membrane were evaluated using quantitative nanomechanical atomic force microscopy (AFM QNM), which allows for precise measurements of Young’s modulus. The sample was probed at multiple points, and the resulting mapping were compiled into a histogram (Figure 7d). The Young’s modulus values ranged from 20 to 80 MPa, with a peak at approximately 43 MPa (see Supporting Information, Figure S18). This Young’s modulus of 43 MPa positions M1@P3 within the range of softer synthetic foams and some rubbers, making it considerably more flexible than conventional hard polymers or resins, yet stiffer than biological tissues such as skin or muscle. Importantly, this level of mechanical strength meets the minimum requirements for all-solid-state polymer electrolytes in lithium-ion batteries (LIBs). A recent review noted that a successful electrolyte must have a Young’s modulus greater than 30 MPa [19]. Furthermore, the mechanical strength values obtained for PILs/poly(propylene carbonate)/LiTFSI membranes are comparable to those reported by other studies [30,38–41].

**Figure 8.**
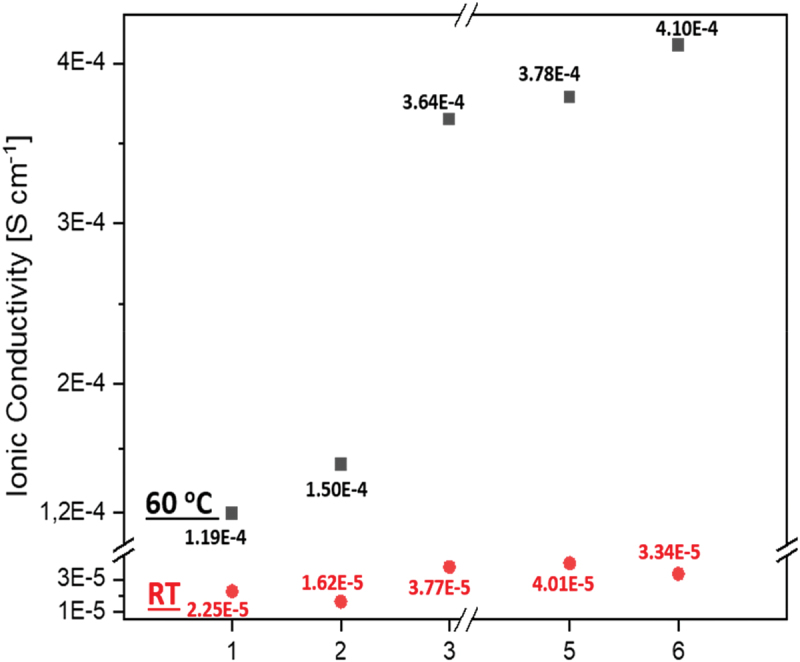
The ionic conductivity results of cross-linked SPEs at RT (red circles) and 60°C (black squares).

Figure 8 displays the ionic conductivity results of the cross-linked membranes at room temperature (RT) and 60°C. The ‘blank’ and LiTFSI-filled N1 networks exhibited the lowest conductivities in the 10^−5^ S·cm^−1^ range at RT, with only a modest increase at 60°C. In contrast, incorporating P3 into the network significantly enhanced conductivity with all P3-based compositions achieving conductivities in the 4 × 10^−4^ S·cm^−1^ range at 60°C. Interestingly, while a substantial increase in LiTFSI content resulted in a slight increase in conductivity, for all non-cross-linked polymers, increasing the salt content to 10 w/w% (corresponding to ~20 mol%) caused an one-order-of-magnitude drop in conductivity to the 10^−5^ S·cm^−1^ level (see Figure 5a).

The results clearly demonstrate the positive impact on conductivity of incorporating the linear polymer P3 into the networks. The similarity in conductivities between the composite N1@P3&LiTFSI (#3, 5, 6) and the corresponding non-cross-linked compositions based on linear polymers (P3&LiTFSI, Figure 5a) suggests that ion conduction in N1@P3&LiTFSI is predominantly provided by the linear polymer, even so that it is a minority component.

The good conductivity observed in the networks with entrapped linear polymer can be attributed to the unrestricted segmental mobility of the polymer chains, which facilitates ion transport. Ion conduction along linear polymer chains occurs more efficiently than along cross-linked segments. The polymer network N1 is composed of relatively short polymer fragments located between adjacent knots, with a mesh size previously estimated to be approximately 3 nm [33]. For the linear polymer P3, the mean chain length can be estimated from its molecular weight (41.8 kg/mol) and corresponding polymerization degree (DP = 60), yielding a contour length of about 15 nm. Thus, the linear polymer provides significantly more extended pathways for unimpeded ion conduction compared to the network, which, according to our hypothesis, accounts for the facilitated ion transport in N1@P3&LiTFSI.

**Figure 9.**
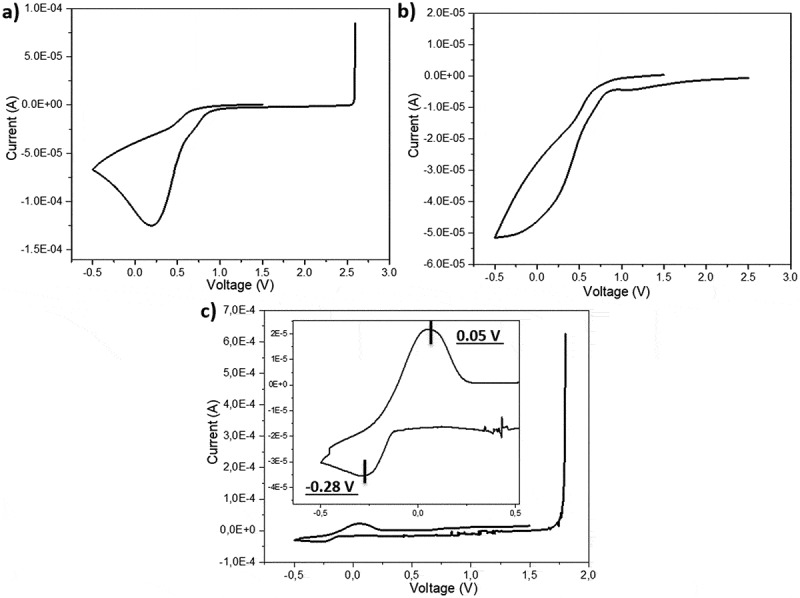
Cyclic voltammetry curves of the lithium plating-stripping experiment for membranes with the compositions: (a) N1@P3_0.13_&LiTFSI_0.08_ (#3); (b), N1@P3_0.26_&LiTFSI_0.16_ (#5); and (c) N1_0.025_@P3_0.26_&LiTFSI_0.26_ (#6) performed at 60°C.

Finally, the electrochemical behavior of the N1@P3_3_&LiTFSI membranes with varying compositions was assessed in lithium plating-stripping experiments conducted in a Swagelok cell at 60°C (see Figure 9a–c).

The N1@P3_0.13_&LiTFSI_0.08_ (#3) membranes exhibited a reduction peak; however, the oxidation peak around 0 V was not observed. A similar behavior was noted for the N1@P3_0.26_&LiTFSI_0.16_ (#5) formulation, which contained twice the amount of polymer and LiTFSI. In contrast, formulation #6 (N1_0.025_@P3_0.26_&LiTFSI_0.26_), characterized by increased LiTFSI content and reduced cross-linking density, displayed both reduction and oxidation peaks at −0.28 V and 0.05 V, respectively. This result highlights the importance of fine-tuning the composition to optimize redox processes in lithium batteries.

## Conclusions

Cross-linking is one of the most straightforward approaches to enhance the mechanical properties of polymer materials, as it forms covalent bonds between polymer chains, creating robust networks. However, when applied to polymer-based electrolytes for lithium batteries, this approach often compromises ionic conductivity. This occurs due to disruptions in the extended conductive pathways and reduced segmental mobility, both of which are crucial for efficient ion transport. To address the trade-off between conductivity and mechanical strength in a cost-effective manner, this study presents the design of a semi-interpenetrating network – three-dimensional polymer network that incorporates a linear ion-conductive polymeric ionic liquid (PIL) containing quaternized imidazolium groups and bis(trifluoromethanesulfonyl)imide anions (LiTFSI).

While pure linear PILs are liquids, their incorporation into cross-linked networks yields rubber-like (which means, much stiffer) materials with a Young’s modulus ranging from 40 to 50 MPa, as measured by quantitative nanomechanical atomic force microscopy. These mechanical properties meet the critical requirement for battery separators, which demand a modulus exceeding 30 MPa. Consequently, mechanically robust, free-standing membranes suitable for lithium battery applications were successfully fabricated.

A particularly notable observation was the different behavior of the polymer-based mixtures containing LiTFSI with respect to salt concentration. For free P3, increasing the LiTFSI concentration from 0 mol% to ~20 mol% led to a tenfold decrease in ionic conductivity. By contrast, when P3 was incorporated into the cross-linked network (N1@P3&LiTFSI), increasing the LiTFSI concentration caused an increase in the conductivity. This result underscores the positive impact of incorporating the linear polymer P3 into the network, preserving high conductivity levels even at increased salt concentrations.

The similar conductivities observed in the composites N1@P3&LiTFSI (#3, #5, #6) compared to their non-cross-linked counterparts (P3&LiTFSI) suggest that ionic conduction in the composite systems is primarily driven by the linear polymer, despite it being a minority component. This can be attributed to the extended pathways provided by the linear PILs, which allow for more efficient ion transport than the shorter, restricted paths in cross-linked networks. This effect is particularly pronounced in oligoethylene oxide-based polymers, highlighting a key finding of this study: the inclusion of oligoethylene oxide chains in polyionic liquids significantly enhances the performance of polymeric solid-state electrolytes. Hence, the mechanically reinforced membranes developed in this work, based on PILs incorporated into polymer networks, hold significant promise as high-performance electrolytes for solid-state batteries.

## Supplementary Material

DMP_242138716_voit_kiriy_SI_revision.docx

## Data Availability

Additional raw data of measurements are available on reasonable request.
