# Supplementary material for: Mechanically stable polymer networks incorporating polymeric ionic liquids for enhanced conductivity in solid-state electrolytes
Source: Des Monomers Polym. 2025 Jan 7;28(1):35–47. doi: 10.1080/15685551.2024.2449444 (PMC11721619; doi:10.1080/15685551.2024.2449444)
Supplement: DMP_242138716_voit_kiriy_SI_revision.docx [file TDMP_A_2449444_SM4265.docx]

**Supporting Information**

**Mechanically Stable Polymer Networks Incorporating Polymeric Ionic Liquids for Enhanced Conductivity in Solid-State Electrolytes**

Sezer Özenler ^a^, Nataliya Kiriy* ^a^, Upenyu L. Muza ^a^, Martin Geisler,^a^ Anton Kiriy ^c^, Brigitte Voit ^a,b^

^a^ Leibniz-Institut für Polymerforschung Dresden e.V., Hohe Str. 6, Dresden 01069, Germany

^b^ Organic Chemistry of Polymers, Technische Universität Dresden, Dresden 01062, Germany

^c^ beeOLED® GmbH Niedersedlitzer Str. 75c, 01257 Dresden, Germany

**Synthesis.**

***Synthesis of M1 and P1.***

The synthesis of M1 and P1 is described elsewhere (see references [31] in the main text).


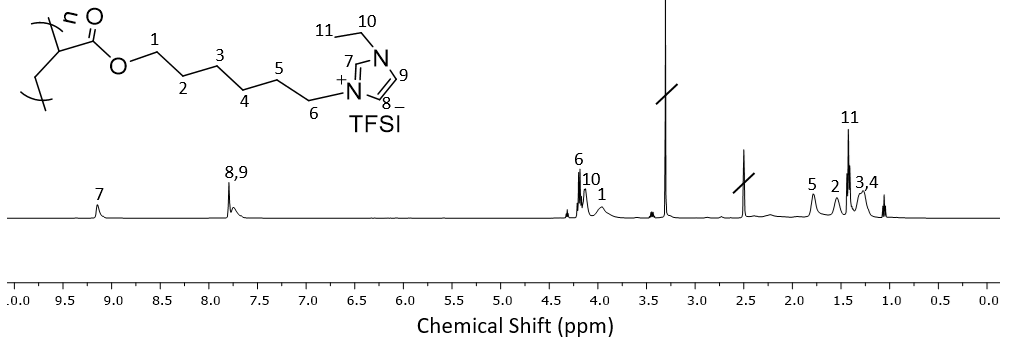
**Figure S1.** ^1^H NMR spectrum of P1.

**Figure S2.** Synthesis of P2.

***Synthesis of Compound 1.***

Potassium hydroxide (5.464 g, 2.2 eq, assay 85%) and imidazole (2.587 g, 1 eq, assay 99%) were added to the flask. Two-necked flask with the condenser and stirrer was connected to the Schlenk line and filled with the Ar (3 evacuate-refill cycles). The 65 ml dry acetonitrile was injected into the flask. The reaction mixture was stirred for a while and followed by dropwise addition of 1-(2-Bromoethoxy)-2-(2-methoxyethoxy)ethane (10 g, 1.1 eq) under nitrogen atmosphere. The reaction the mixture was refluxed at 80 °C under stirring for 6 h. After filtration and evaporation of acetonitrile, the product dissolve in dichloromethane (200 mL), washed with water (3 × 50 mL). The purity is 90% and the yield is 93% (DOI: 10.1007/s10570-022-04883-1). The ^1^H NMR (500 MHz, DMSO-*d6*): δH (ppm) = 7.58 (s, 1H), 7.15 (s, 1H), 6.86 (s, 1H), 4.10 (t, 2H), 3.67 (t, 3H), 3.50 (m, 6H), 3.42 (t, 2H), 3.24 (s, 3H).


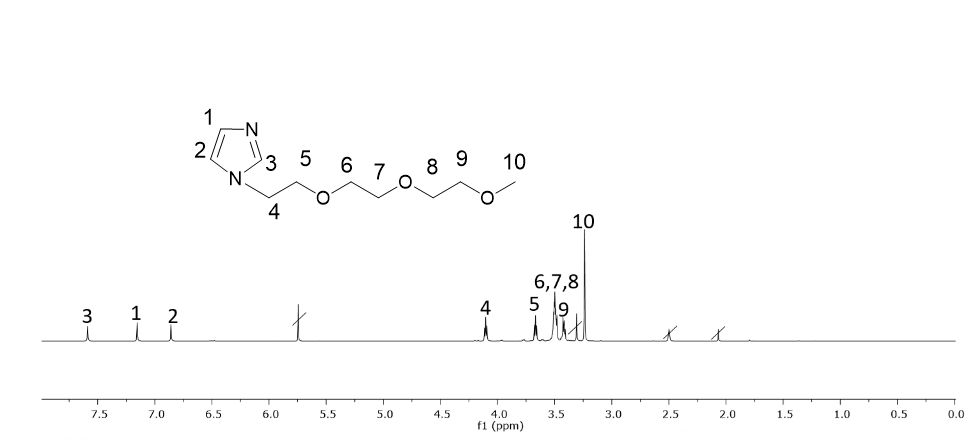


**Figure S3.** ^1^H NMR spectrum of Compound 1.

***Synthesis of Compound 2.***

The synthesis of Compound is described elsewhere (DOI: 10.1002/macp.202200317). The ^1^H NMR see Figure S3.


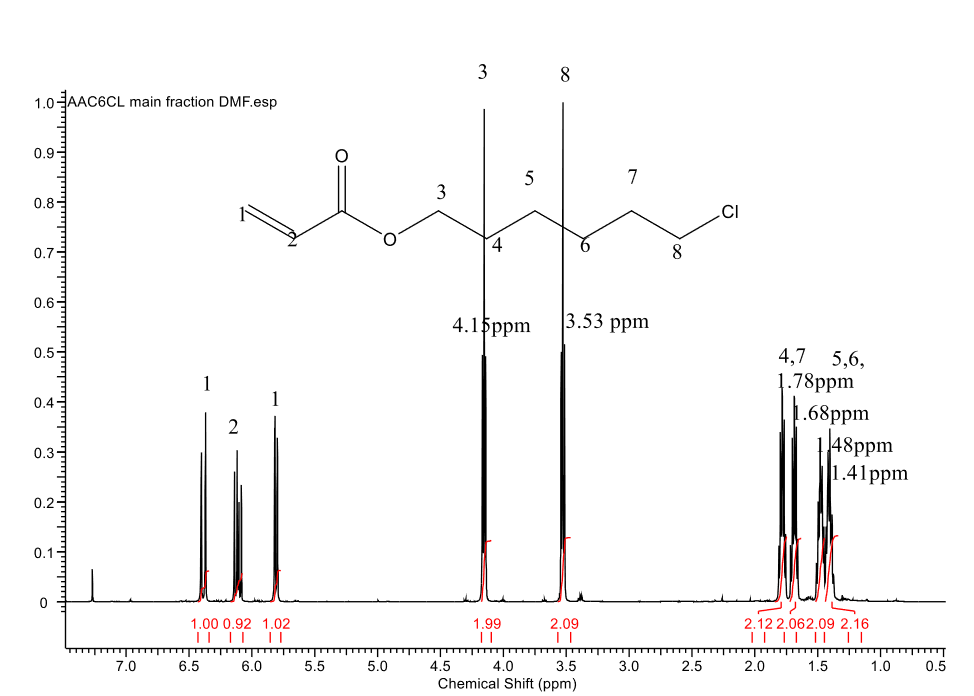


**Figure S4.** ^1^H NMR spectrum of Compound 2

***Polymerization of Compound 3.***

Compound 2 (3.0 g, 1 eq) and azobisisobutyronitrile (AIBN) (51 mg, 0.02 eq) were dissolved in 50 ml of ethanol absolute. The O_2_ from the mixture was removed by 3-5 freeze/thaw cycles. Then the flask was filled with the argon. After that the reaction mixture was heated up to 70 °C and stirred for 24 hours under Ar with reflux condenser. After reaction medium was cooled down, and ethanol decanted. Add 10 ml ethanol stirred 1 hours with following decantation three times. The yield is 65%. The ^1^H NMR (500 MHz, DMSO-d6): δH (ppm) = 6.31 (dd, 1H), 6.16 (dd, 1H), 5.94 (dd, 1H), 4.15 (t, 2H), 3.53 (t, 2H), 1.78 (quin, 2H), 1.68 (quin, 2H), 1.48 (quin, 2H), 1.41 (quin, 2H).

**Figure S5.** ^1^H NMR spectrum of Compound 3

***Synthesis of Compound 4 and P2.***

Compound 3 (1.0 g, 1 eq) and EtOX-IM (1.21 g, 1.1 eq) were heated up to 75 °C and stirred for 6 days. Acetonitrile was added when the stirrer couldn’t rotate due to high viscosity. The conversion is 97% and the yield is 100%. The reaction was monitored by ^1^H NMR. The anion exchange of Compound 4 with TFSI^-^, which included the next step, was continued without purification due to the unreacted of the adduct. Compound 4 (2.1014 g, 1 eq) was dissolve in 30 ml DI water and was heated up to 60 °C. LiTFSI (2,090 g, 1,4 eq) was dissolved in 25 ml water solution and dropwise added. As soon as added LiTFSI solution, the clear reaction medium become non-transparent. After 2 hours precipitation was occurred. After 17 hours, reaction was cooled down, and DI water decanted. Precipitation washed 3 time with DI water. The polymer P2 was obtained pure, and well-defined with ^1^H NMR. The yield is 70%.


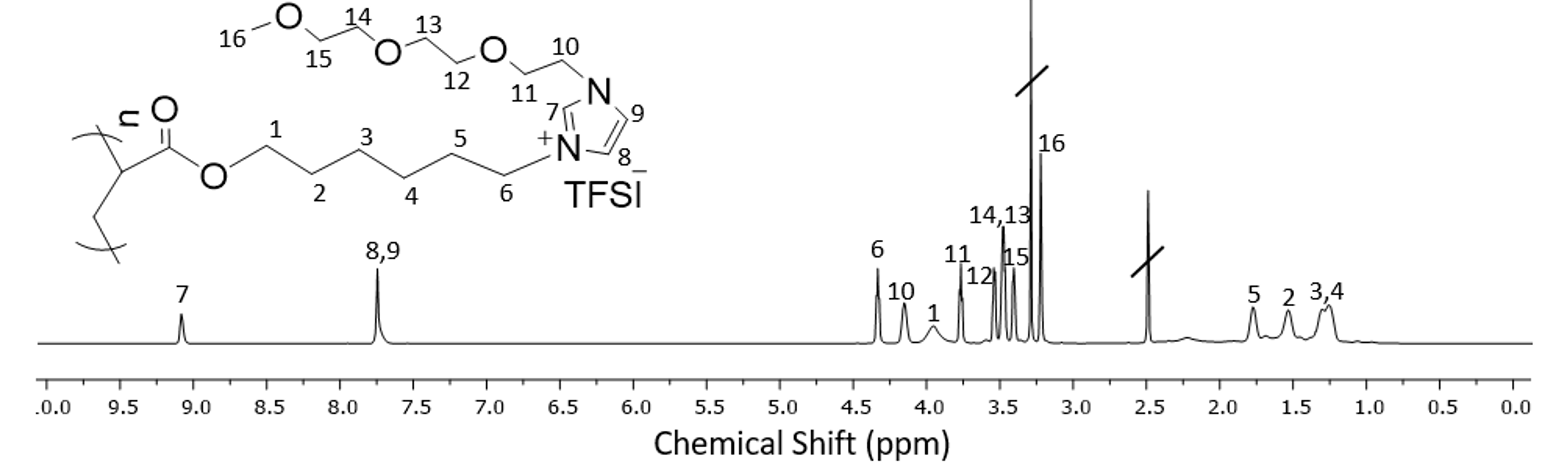
**Figure S6.** ^1^H NMR spectrum of P2.

**Figure S7.** Synthesis of P3.

***Synthesis of Compound 6.***

Two-necked flask (capacity 250 ml) with the condenser and stirrer was connected to the Schlenk line and filled with the Ar (3 evacuate-refill cycles). The 75 ml of the solvent (THF) were injected into the flask. Then the 2-[2-(2-chloroethoxy)ethoxy]ethanol was weighed in the syringe and added to the flask (96%, 9.13 g, 0.052 mol, 1 eq). The exact amount of the added substance was determined by weighting the syringe after injection. On the basis of this value the amount of triethylamine was calculated and added to the solution (99%, 10.57 g, 0.103 mol, 2 eq). The reaction mixture was stirred for 1 hour at room temperature. Next the flask was cooled down to 0 °C, the acryloyl chloride (97%, 5.78g, 0.062 mol, 1.2 eq) was added dropwise in 25 ml dry THF (to avoid warming up the reaction mixture which can lead to the polymerization), and the solution was stirred for 10 minutes. During this process the formation of the white precipitate was observed. The reaction mixture was stirred for 24 hours at 45 °C. After filtration and evaporation of THF, the product dissolve in chloroform (100 ml), washed with water (3 × 100 ml). The compound 6 was obtained with a yield 91%. The ^1^H NMR (500 MHz, DMSO-*d6*): δH (ppm) = 6.34 (dd, 1H), 6.20 (dd, 1H), 5.95 (dd, 1H), 4.23 (t, 2H), 3.69 (m, 6H), 3.58 (m, 4H).

**
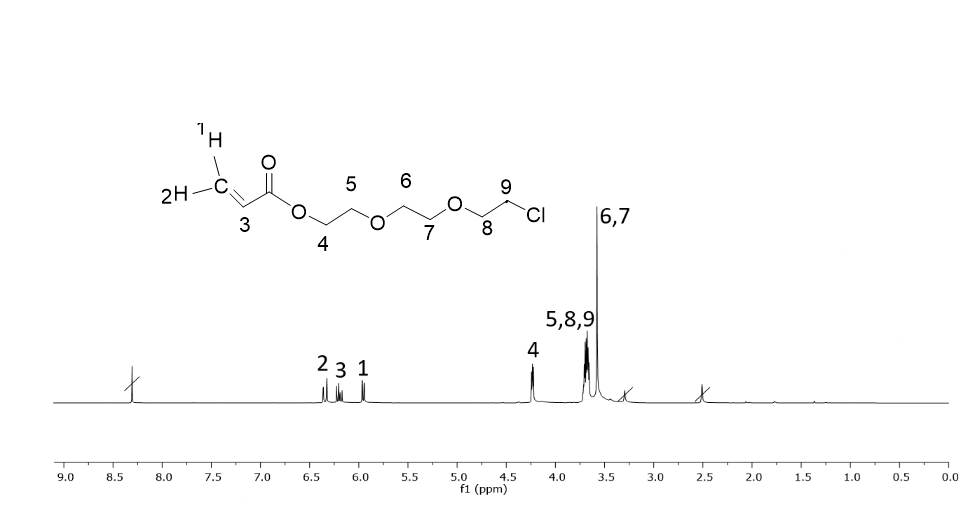
Figure S8.** ^1^H NMR spectrum of Compound 6.

***Synthesis of Compound 7.***

9,74 g of Compound 6 (43,86 mmol) of the monomer and 0,144 g AIBN (0,877 mmol) were dissolved in 250 ml of ethanol absolute. The O_2_ from the mixture was removed by 4 freeze/thaw cycles. After that the reaction mixture was heated up to 80 °C and stirred for 24 hours under Ar with reflux condenser. Reaction medium was cooled down, and ethanol decanted. Washed with ethanol and decantation three times. The yield is 57%.

**
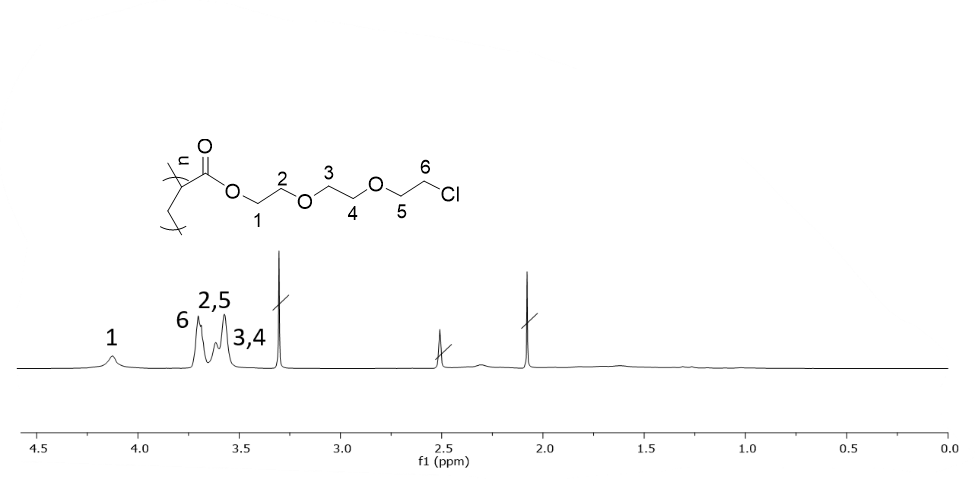
Figure S9.** ^1^H NMR spectrum of Compound 7.

***Synthesis of Compound 8 and P3.***

Compound 7 (2.0 g, 1 eq) and compound 1 (2.10 g, 1.1 eq) were heated up to 75 °C and stirred for 7 days. Acetonitrile was added when the stirrer couldn’t rotate due to high viscosity. The reaction was monitored by 1H NMR. The conversion is 93% and the yield is 100%. The anion exchange of Compound 8 with TFSI^-^, which included the next step, was continued without purification due to the unreacted of the adduct. Compound 8 (4.121 g, 1 eq) was dissolve in 60 ml DI water and was heated up to 60 °C. LiTFSI (3,797 g, 1,4eq) was dissolved in 50 ml water solution and dropwise added. As soon as added LiTFSI solution, the clear reaction medium become non-transparent. After 2 hours precipitation was occurred. After 17 hours, reaction was cooled down, and DI water decanted. Precipitation washed 3 time with DI water. The polymer P3 was obtained pure, and well-defined with ^1^H NMR. The yield of P3 is 70%. The ^1^H NMR


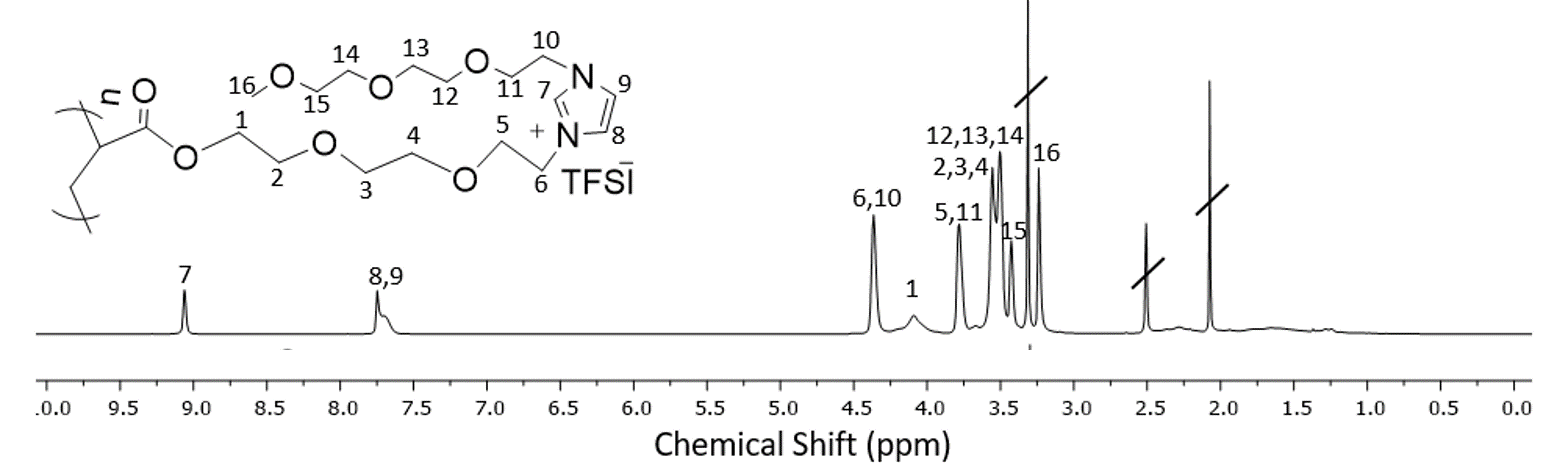
**Figure S10.** ^1^H NMR spectrum of P3.

***ThFFF-MALDI TOF MS***


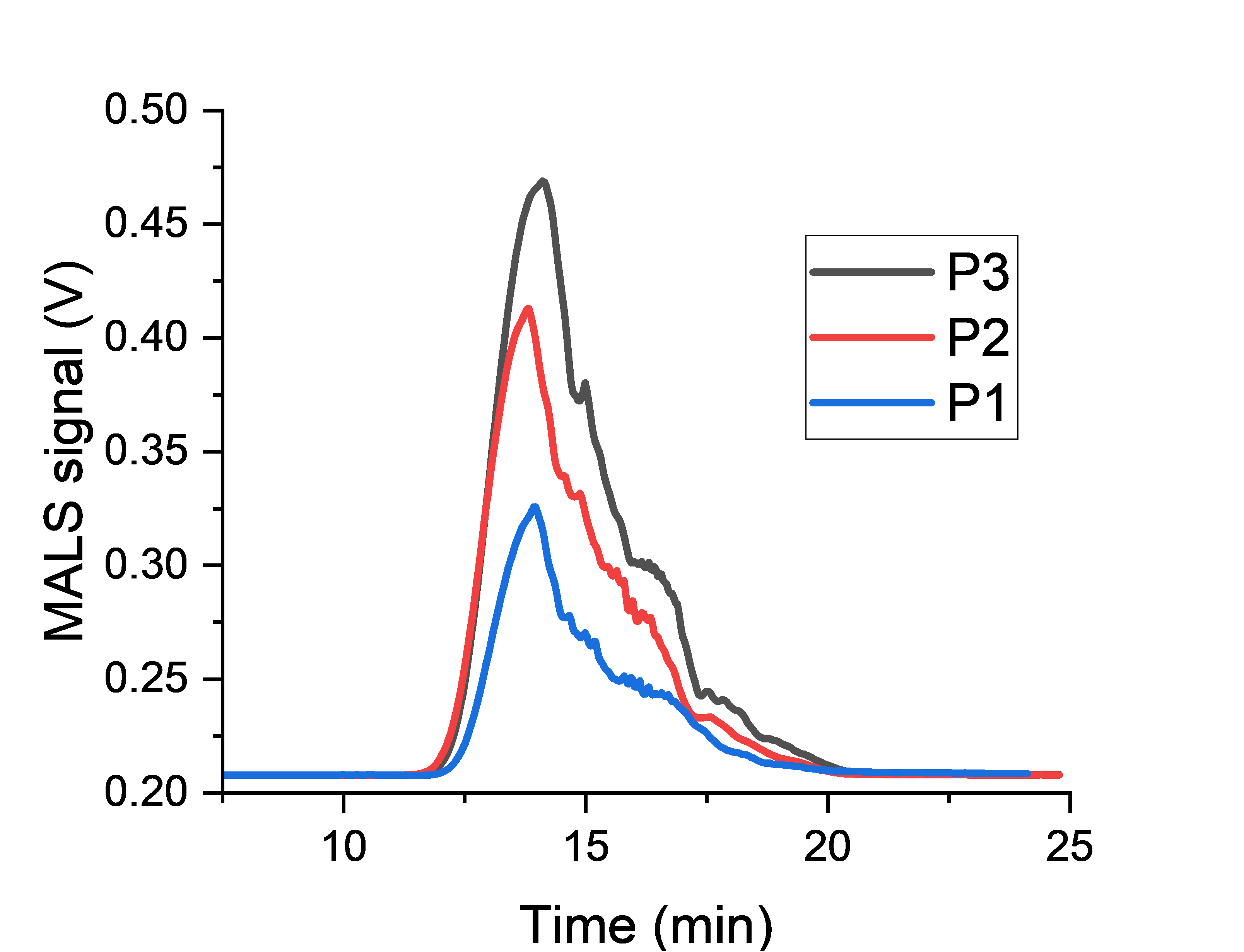


**Figure S11a.** ThFFF fractogram based on the MALS detector signal in voltage units (V)


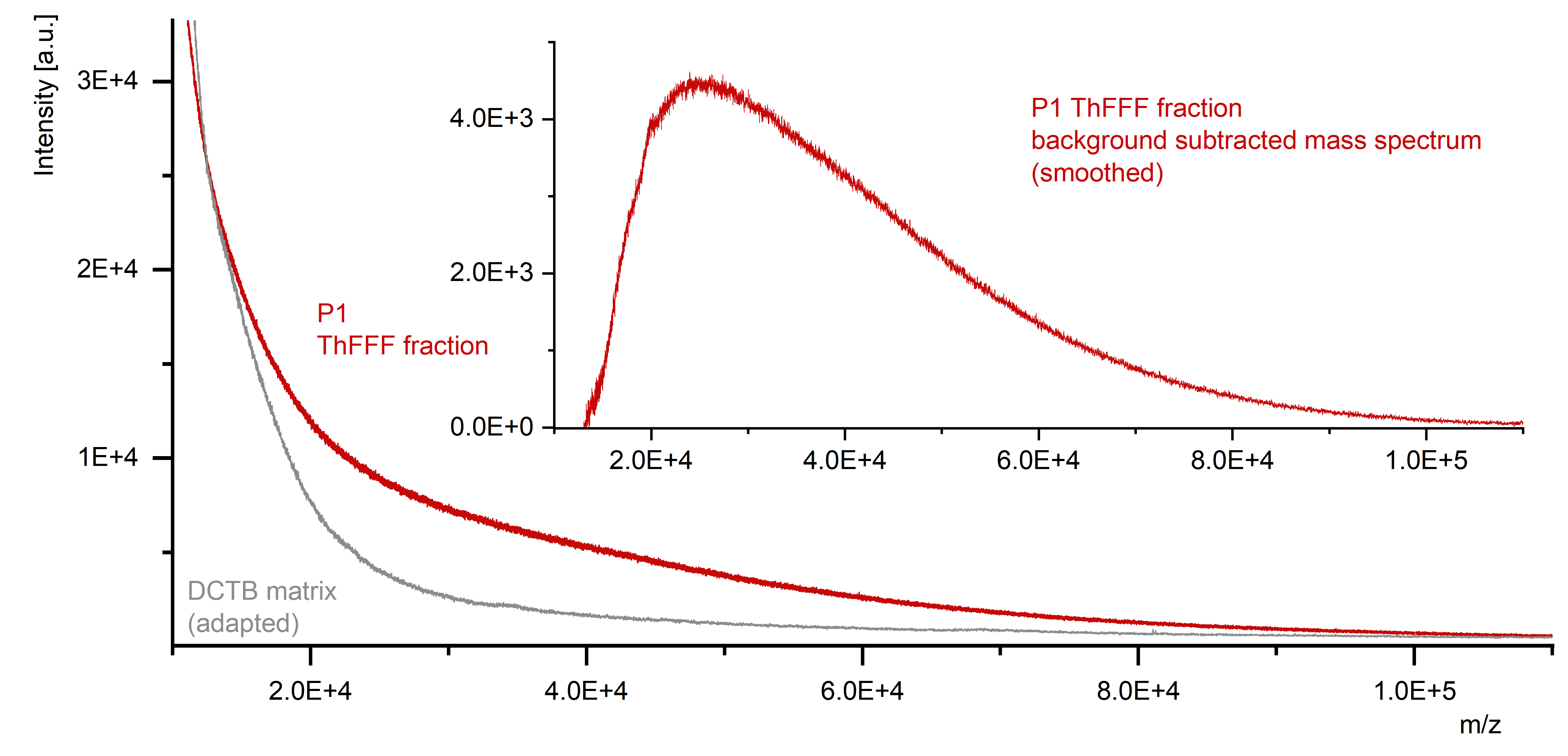


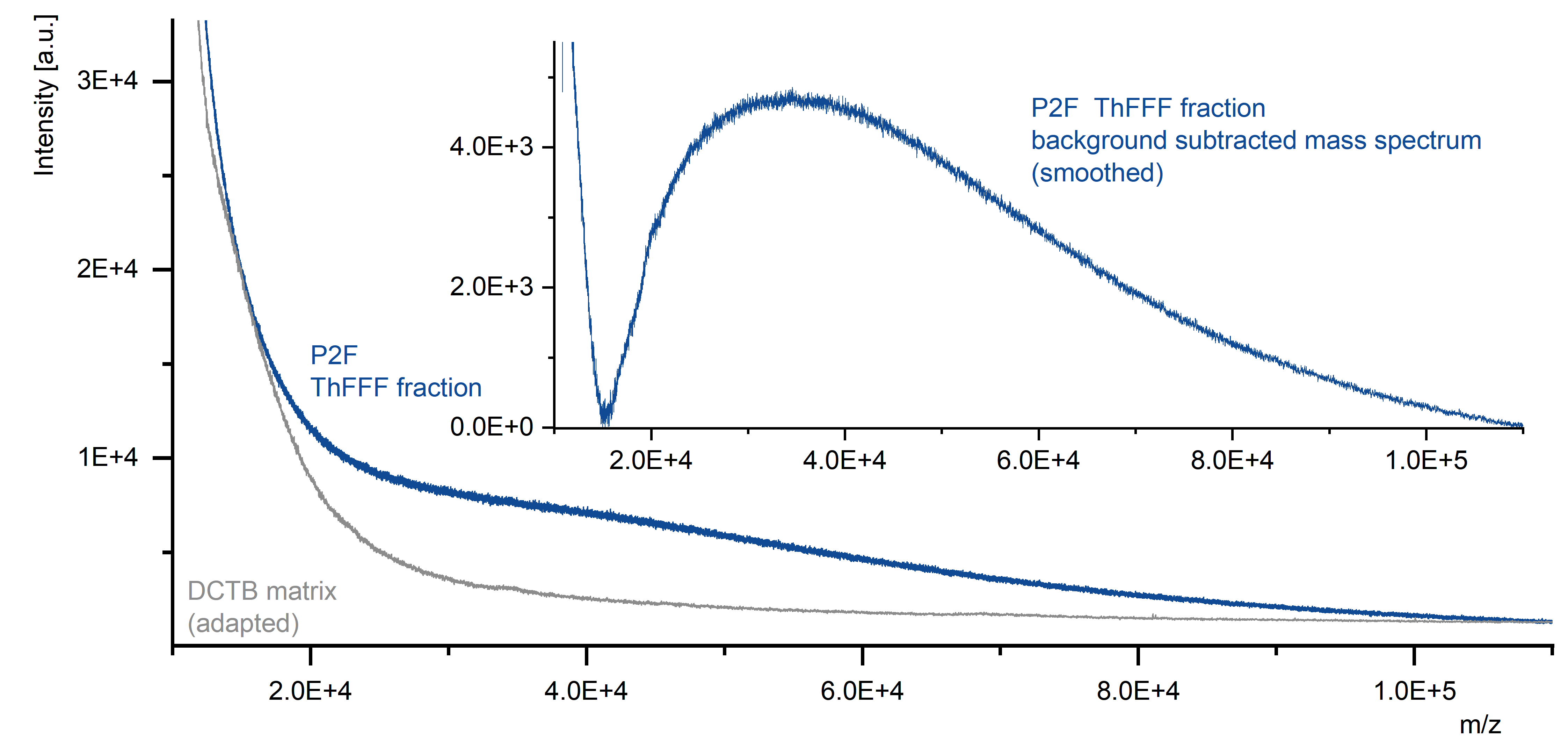


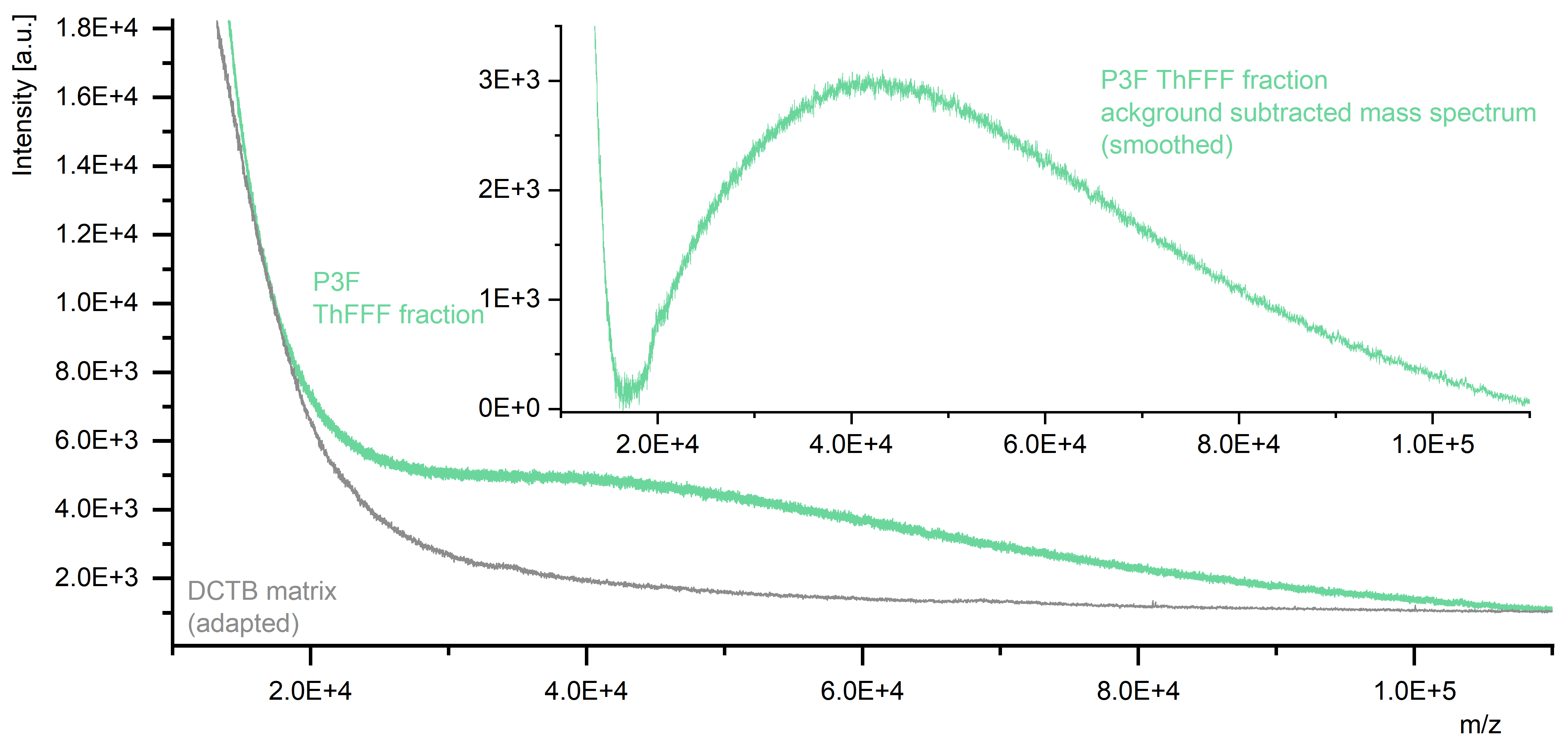


**Figure S11b.** MALDI TOF MS analysis of ThFFF fractions

**Table S1.** Summary of average molar masses of the sample in total and of the deconvoluted mass peak z 1

| Sample | *M_n_* [g/mol] | | | *M_w_* [g/mol] | | | *Ð* (*M_n_*/*M_w_*) | | |
| --- | --- | --- | --- | --- | --- | --- | --- | --- | --- |
| P1F | 30700 | ± | 1800 | 36300 | ± | 3200 | 1.19 | ± | 0.02 |
| P2F | 36700 | ± | 1600 | 43500 | ± | 1800 | 1.185 | ± | 0.004 |
| P3F | 41800 | ± | 1800 | 48700 | ± | 1900 | 1.165 | ± | 0.006 |

**Study of thermal properties.**

a

b

c

**Figure S12.** DSC results: **(**a) P1, (b) P2, (c) P3.

**Table S2.** TGA results of the P1, P2 and P3. (IDT: the initial decomposition temperature; Td10%: the temperature at which 10% weight loss occurs; Td50%: the temperature at which 50% weight loss occurs, MDT: the maximum decomposition rate temperature.

| PIL | IDT [°C] | Td10% [°C] | Td50% [°C] | MDT [°C] |
| --- | --- | --- | --- | --- |
| P1 | 305 | 360 | 395 | 420 |
| P2 | 300 | 380 | 410 | 425 |
| P3 | 303 | 350 | 390 | 430 |

**Measurement of ionic conductivity.**

**
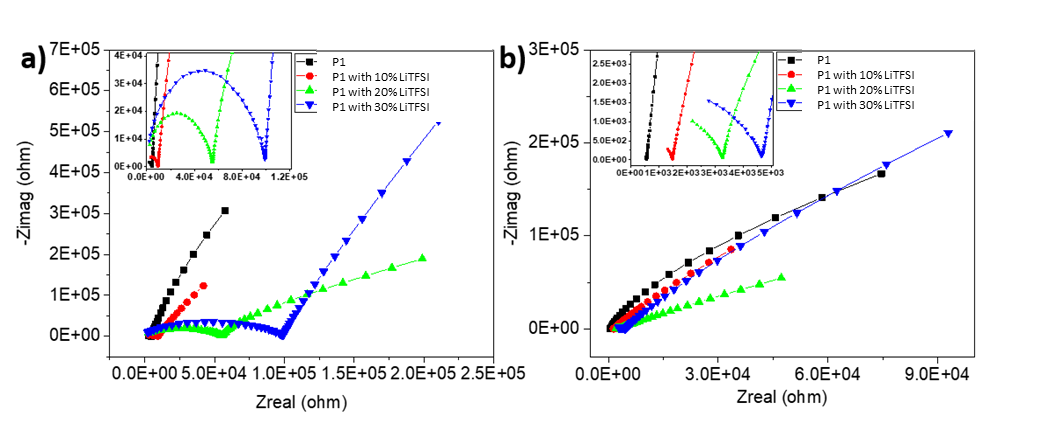
**

**Figure S13.** Nyquist plots of the P1 with additional 0, 10, 20, and 30 w/w% LiTFSI a) at RT b) at 60 °C

**
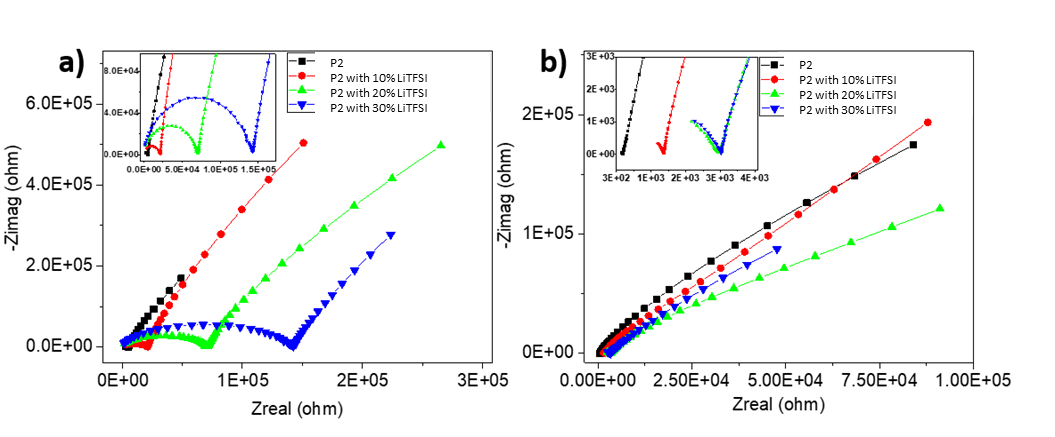
**

**Figure S14.** Nyquist plots of the P2 with additional 0, 10, 20, and 30 w/w% LiTFSI a) at RT b) at 60 °C

**
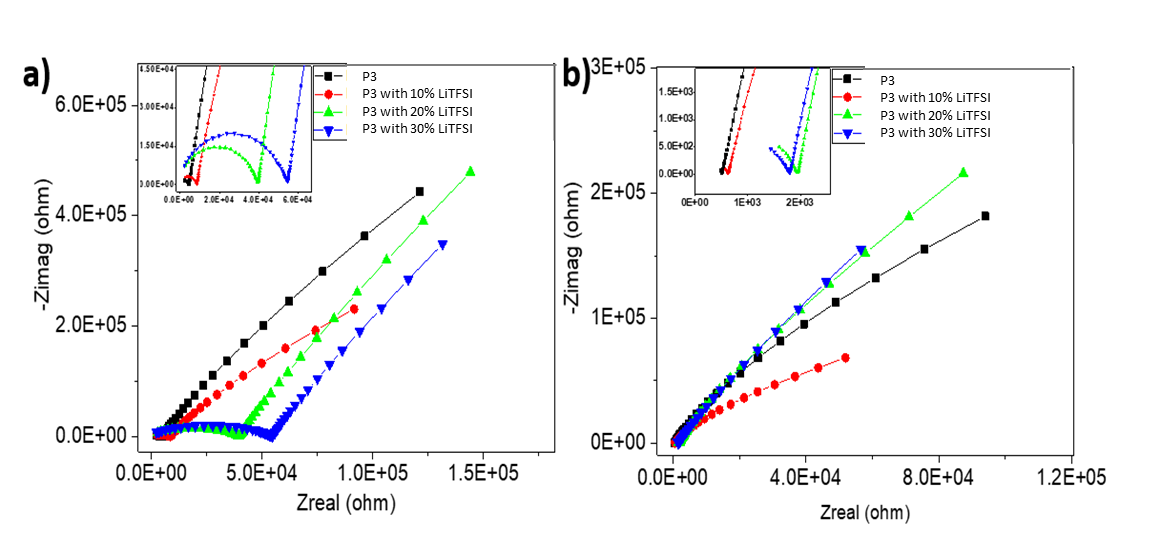
**

**Figure S15.** Nyquist plots of the P3 with additional 0, 10, 20, and 30 w/w% LiTFSI a) at RT b) at 60 °C

**
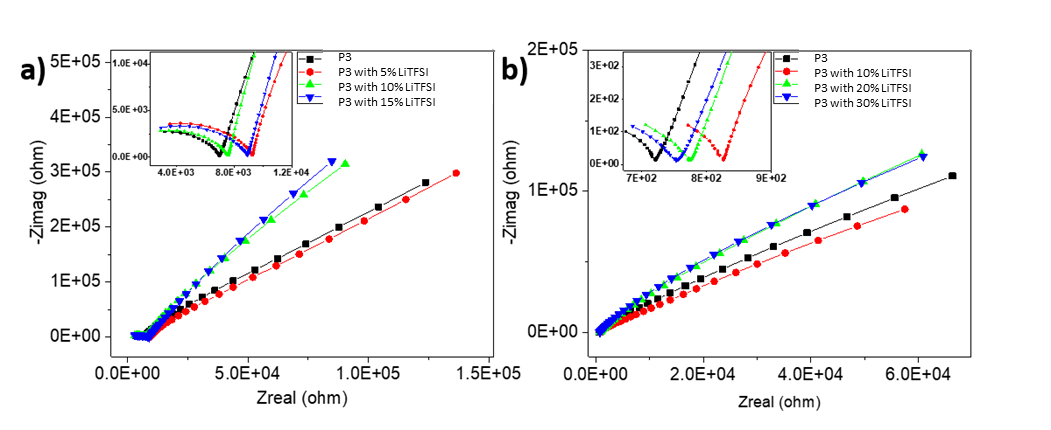
**

**Figure S16.** Nyquist plots of the P3 with additional 0, 5, 10, and 15 mol% LiTFSI a) at RT b) at 60 °C

**
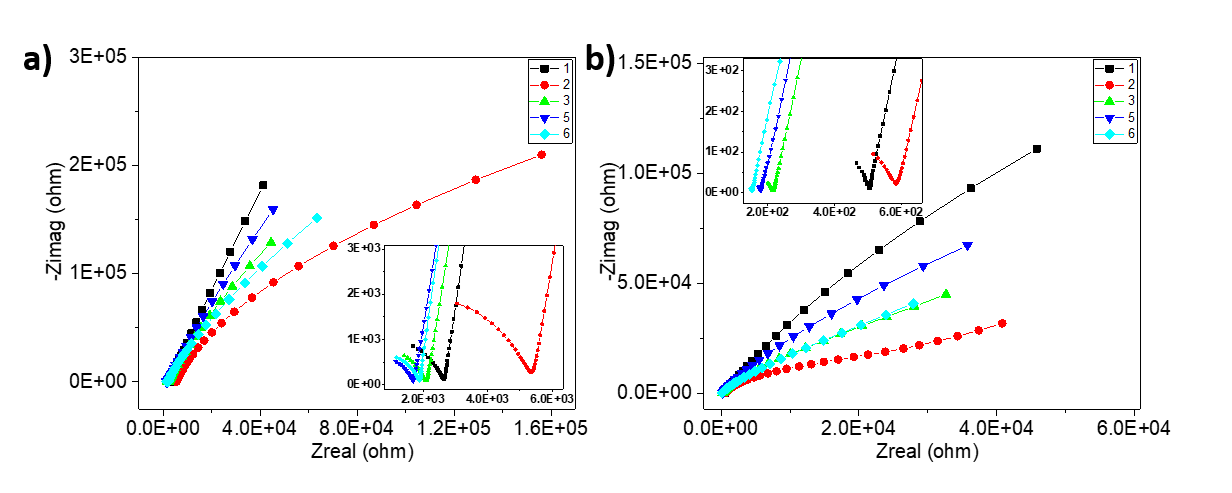
Figure S17.** Nyquist plots of the SPEs a) at RT, b) at 60 °C, for the following networks: N1(1), N1@&LiTFSI_0.1_ (2), N1@P3_0.13_&LiTFSI_0.08_ (3), N1@P3_0.15_ (4), N1@P3_0.26_&LiTFSI_0.16_ (5), and N1_0.025_@P3_0.26_&LiTFSI_0.26_ (6).

**Measurement of the transference number.**

**Table S3.** Measured of the transference number of N1@P3&LiTFSI ^a^

| *I_0_*(A) | *I_ss_*(A) | *I_ss_*/ *I_0_* | *R_0_*(ohm) | *Rss*(ohm) | $\Delta V$(mV) | *t*_+_ |
| --- | --- | --- | --- | --- | --- | --- |
| 1.209E-4 | 2.275E-5 | 0.19 | 361 | 374 | 50 | 0.03 |

^a^ The space of electrodes is 0.1 mm.

**Measurement of Young Modulus.**

**
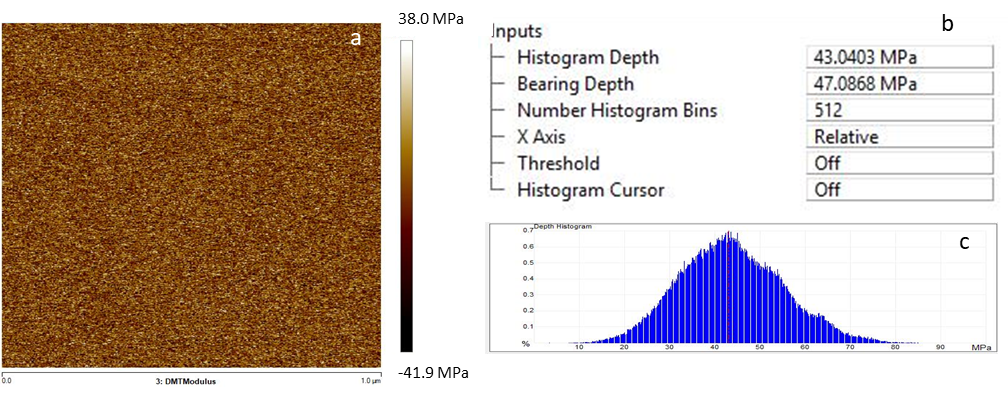
**

**Figure S18.** AFM Quantitative nanomechanical analysis of the AN1@P3&LiTFSI membrane: (a) DMT Modulus image; (b) Modulus histogram analysis; (c) Modulus histogram.

**1.7 E-4**

**Figure S19.** (a) Influence of added LiTFSI on the ionic conductivity of P1, P2, and P3 at room temperature (RT) and 60 °C with LiTFSI content given in mol% (corresponds to **Figure 5a** of the main text, which gives data for the LiTFSI content given in w/w%).
